# IL-10-Dependent and -Independent Mechanisms Are Involved in the Cardiac Pathology Modulation Mediated by Fenofibrate in an Experimental Model of Chagas Heart Disease

**DOI:** 10.3389/fimmu.2020.572178

**Published:** 2020-09-24

**Authors:** Jimena Rada, Martín Donato, Federico N. Penas, Catalina Alba Soto, Ágata C. Cevey, Azul V. Pieralisi, Ricardo Gelpi, Gerardo A. Mirkin, Nora B. Goren

**Affiliations:** ^1^Departamento de Microbiología, Parasitología e Inmunología, Facultad de Medicina, Universidad de Buenos Aires, Buenos Aires, Argentina; ^2^Instituto de Investigaciones Biomédicas en Retrovirus y SIDA, CONICET-Universidad de Buenos Aires, Buenos Aires, Argentina; ^3^Departamento de Patología, Facultad de Medicina, Universidad de Buenos Aires, Buenos Aires, Argentina; ^4^Instituto de Fisiopatología Cardiovascular, CONICET-Universidad de Buenos Aires, Buenos Aires, Argentina; ^5^Instituto de Investigaciones en Microbiología y Parasitología Médica, CONICET-Universidad de Buenos Aires, Buenos Aires, Argentina

**Keywords:** *Trypanosoma cruzi*, IL-10 knockout mice, fenofibrate, chronic chagasic cardiomyopathy, inflammatory response

## Abstract

IL-10 is an anti-inflammatory cytokine that plays a significant role in the modulation of the immune response in many pathological conditions, including infectious diseases. Infection with *Trypanosoma cruzi* (*T. cruzi*), the etiological agent of Chagas disease, results in an ongoing inflammatory response that may cause heart dysfunction, ultimately leading to heart failure. Given its infectious and inflammatory nature, in this work we analyzed whether the lack of IL-10 hinders the anti-inflammatory effects of fenofibrate, a PPARα ligand, in a murine model of Chagas heart disease (CHD) using IL-10 knockout (IL-10 KO) mice. Our results show fenofibrate was able to restore the abnormal cardiac function displayed by *T. cruzi-*infected mice lacking IL-10. Treatment with fenofibrate reduced creatine kinase (CK) levels in sera of IL-10 KO mice infected with *T. cruzi.* Moreover, although fenofibrate could not modulate the inflammatory infiltrates developing in the heart, it was able to reduce the increased collagen deposition in infected IL-10 KO mice. Regarding pro-inflammatory mediators, the most significant finding was the increase in serum IL-17. These were reduced in IL-10 KO mice upon fenofibrate treatment. In agreement with this, the expression of RORγt was reduced. Infection of IL-10 KO mice increased the expression of YmI, FIZZ and Mannose Receptor (tissue healing markers) that remained unchanged upon treatment with fenofibrate. In conclusion, our work emphasizes the role of anti-inflammatory mechanisms to ameliorate heart function in CHD and shows, for the first time, that fenofibrate attains this through IL-10-dependent and -independent mechanisms.

## Introduction

Infection with *Trypanosoma cruzi* (*T. cruzi*), the etiological agent of Chagas disease, triggers both innate (1–3), and adaptive (4–6) immune responses that aim at the control of the parasite load both in tissues and peripheral blood [Reviewed in (7)]. However, these mechanisms do not succeed in the complete eradication of the parasite, which results in parasite persistence (8–12).

An ongoing inflammatory process dysregulated by yet unknown factors, associated with unsuccessful parasite control, leads to severe consequences in 30–40% of infected people late during the chronic phase of which Chronic Chagasic Cardiomyopathy (CCC), a form of dilated cardiomyopathy, is the most frequent consequence (13, 14). Its main features are the development of cardiac conduction system anomalies leading to arrhythmia, microvascular alterations leading to thromboembolism, and inflammatory processes that promote heart muscle fibrosis. These patients may undergo heart chamber remodeling, congestive heart failure and death (15, 16).

The inflammatory response is characterized by the recruitment of mononuclear cells to the heart and other tissues, and increased systemic and local inflammatory mediators, including cytokines like IL-1β, IL-6, and TNFα and enzymes like iNOS, involved in the production of NO, or matrix metalloproteinases like MMP-9, that contribute to tissue remodeling (17–19). Despite the low levels of parasite load observed during the chronic phase of infection, its persistence plays a crucial role to uphold the inflammatory reaction leading to heart anomalies and remodeling (10, 20).

In this regard, the balance between the pro-inflammatory/pro-fibrotic cytokine IL-17, mainly produced by Th17 cells (21, 22), and the anti-inflammatory/anti-fibrotic cytokine IL-10, produced by Treg, alternatively activated macrophages and CD5 B cells (23–25), should be considered. Overall, persistent expression of IL-17 would promote fibrosis and tissue scar formation in several pathological conditions, including systemic sclerosis (26), kidney fibrosis in diabetes (27), primary biliary cirrhosis (28), and ischemic heart failure involving ventricular arrhythmia (29). Conversely, IL-10 has been shown to promote the senescence of hepatic stellate cells *via* STAT3/p53, in a model of Cl_4_C-induced fibrosis in rats (30), reduce fibrosis during cutaneous wound healing in human and mice (31), and ameliorate high-fat-diet atrial remodeling in C57Bl/6 IL-10 KO mice, preventing fibrillation (32). Importantly, the regulation of M2 macrophage polarization by IL-10 seems to be critical to preclude the harmful outcome of scar formation in different tissues (33, 34).

Considering the role of IL-10 in CCC, a recent work shows that a betulinic acid derivative is able to reduce inflammation and fibrosis, associated with a significant increase in serum IL-10 levels, in an experimental model of chronic Chagas disease. However, these authors do not report any changes in the electrocardiographic or ergometry registers resulting from treatment (35). Interestingly, it has been shown that IL-10 inhibits the production of pro-inflammatory cytokines signaled by the NF-kB and ERK/MAPK pathways through upregulation of SOCS3 in cardiomyocytes infected *in vitro* with *T. cruzi* (36).

Given the inflammatory nature of CCC, pharmacological interventions focused on the control of its deleterious effects may prove successful in ameliorating the course and outcome of the disease. Concerning this, fibrates have emerged as putative anti-inflammatory drugs, besides their effects to control dyslipidemias (37). Remarkably, we recently published evidence that fenofibrate is able to restore heart function, as assessed by echocardiography, and reduce inflammatory mediators expressed in the heart, as well as serum markers of heart damage, in an experimental model of Chagas disease (38). However, the role that fenofibrate may play in the context of the pro/anti-inflammatory imbalance leading to pathology, is still unclear.

In this work we study the consequences of IL-10 depletion on the effects of fenofibrate on cardiac function, proinflammatory response and heart remodeling in a model of Chagas heart disease (CHD) resembling CCC.

## Materials and Methods

### Ethics Statement

Mice used in this study were bred and maintained in the animal facility at the Instituto de Investigaciones en Microbiología y Parasitología Medica, Universidad de Buenos Aires-CONICET. All procedures carried out with mice were approved by the Institutional Committee for the Care and Use of Laboratory Animals (CICUAL, Facultad de Medicina de la Universidad de Buenos Aires) in line with guidelines of the Argentinean National Administration of Medicines, Food and Medical Technology (ANMAT), Argentinean National Service of Sanity and Agrifoods Quality (SENASA) and also based on the US NIH Guide for the Care and Use of Laboratory Animals.

### Mice

To carry out this work, BALB/c-background IL-10 knockout (IL-10 KO) mice, homozygous for the targeted mutation *Il10tm1Cgn* (Stock Number 004333; The Jackson Laboratory, United States) (39) and wild type (WT) BALB/c mice were used.

### Experimental Design

Eight-weeks old BALB/c IL-10 KO and WT male mice were infected by intraperitoneal route with 1 × 10^5^ bloodstream trypomastigotes of the non-lethal K-98 clone of *T. cruzi* (DTU I) (40, 41). At weeks 5 and 9 post-infection (pi), echocardiographic studies were carried out to evaluate cardiac dysfunction. The following scheme depicts the experimental design:


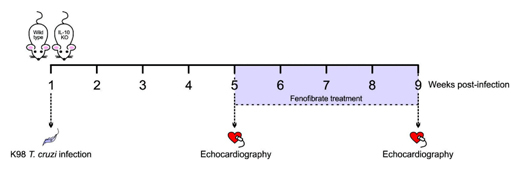


### Treatment

Mice were treated by oral gavage with fenofibrate (100 mg/kg/day) Daunlip^®^, Montpellier S.A, Argentina. PubChem Compound Database CID = 3339, Fen, suspended in phosphate-buffered saline (PBS) for 30 consecutive days, since week 5 pi.

### Parasitemia and Survival

Presence of parasites in blood was evaluated by the microhematocrit method (42) at week 3 post infection (pi). Parasitemia was analyzed weekly, using the method of Pizzi modified by Brener (1962) (43), from week 3 pi until the end of the experiment. Survival was observed daily, until the end of the experiment. Parasitemia was expressed as parasites *per* milliliter.

### Doppler Echocardiography

Transthoracic echocardiography was performed using an Acuson Sequoia C 512 ultrasound system with a 14-MHz linear transducer. Echocardiographic experiments were conducted under light anesthesia (287.5 mg/kg of 2.5% sterile-filtered 2,2,2-tribromoethanol; Sigma-Aldrich).

The two-dimensional parasternal short-axis imaging plane was used to obtain M-mode tracings at the level of the papillary muscles. Left ventricular (LV) internal dimensions and LV wall thickness (LVWT) were determined at systole and diastole using leading-edge methods and the American Society of Echocardiography guidelines (44). End-diastolic measurements were taken at the maximal LV diastolic dimension, and end systole was defined as the time of the most anterior systolic excursion of the posterior wall. Measurements were taken from three consecutive beats for each mouse. Ejection fraction (EF) and shortening fraction (SF) were calculated and used as ejective indexes of systolic function. EF was estimated from LV dimensions by the cubed method as follows: EF (%) = [(LVEDD3-LVESD3)/LVEDD3] × 100, where LVEDD is LV end-diastolic diameter, and LVESD is LV end-systolic diameter. The isovolumic relaxation time (IVRT) was measured by the Doppler-echo study (38).

### Histological Studies

Hearts from untreated uninfected controls, *T. cruzi*-infected and *T. cruzi*-infected and fenofibrate-treated IL-10 KO and WT mice were fixed in PBS-buffered 4% paraformaldehyde and included in paraffin after dehydration. Six non-contiguous sections (5 μm) were stained with hematoxylin-eosin or picrosirius red for the examination of cellular infiltrates and collagen deposits. Images from thirty random microscopic fields (400x) were acquired using an Eclipse E600 microscope (Nikon Inc.) equipped with a Spot RT digital camera. Analysis was performed using the Image J software (NIH, United States) (38).

### Creatine Kinase Activity

Serum creatine kinase (CK) activity was measured in the serum of uninfected, *T. cruzi*-infected, and *T. cruzi*-infected fenofibrate-treated IL-10 KO and WT mice using a colorimetric method based on the photometric NADP reduction assay according to manufacturer’s instructions (Wiener Lab, Rosario, Argentina). Absorbance was measured at 340 nm.

### mRNA Purification

Total RNA was obtained from heart tissue homogenates using Quickzol reagent (Kalium) treated with DNAse (Life Technologies). Total RNA was reverse-transcribed using Expand Reverse Transcriptase (Invitrogen Corp., MA, United States), according to manufacturer’s instructions.

### Quantitative Reverse Transcription Polymerase Chain Reaction

Quantitative reverse transcription polymerase chain reaction (RT-qPCR) was performed using a 5 × HOT FIREPol^®^ EvaGreen^®^ qPCR Mix Plus (ROX; Solis BioDyneCorp., Estonia) in an Applied Biosystems 7500 sequence detector. PCR parameters were 52°C for 2 min, 95°C for 15 min, and 40 cycles of 95°C for 30 s and 60°C (for 18S, IL-6, iNOS and Mannose Receptor), 64°C (for TGF-β and FOXP3), 58°C (for YMI, FIZZ and RORγt), or 62°C (for TNF-α). Quantification was performed using the comparative threshold cycle (Ct) method and the efficiency of the RT reaction (relative quantity, using the 2^–^^ΔΔ^^Ct^ method). Replicates were then averaged, and fold induction was determined considering the value at time 0 as 1 (36).

### Primer Sequences

Primer sequences used in this work were designed following the next protocol:

The database “Nucleotide” (NCBI, NIH, MD, United States) was used to look for the gene to be amplified. Once the complete sequence of the target mRNA was found, it was downloaded in FASTA format.

The sequence was read using the software “Oligo,” v.6 (Molecular Biology Insights, Inc., CO, United States).

Using this software, the following requisites for the custom primers were set:

• Product length: Between 50 to 300 base pairs.
• Melting temperature for the primers: 58–62°C.
• Percentage of GC: >50%, to attain some stability.
• The ΔG formation: 0 to −3.5 Kcal/mol.
• The primers were searched in the middle of the gene.

Duplex and hairpin formation were analyzed with Oligo v.6 software as well. The custom primers did not create neither the duplex nor the hairpin, indicating their good quality.

Once the pair of primers for each amplification was obtained, the specificity was checked in the Genome Browser (www.genome.ucsc.edu, University of California, Santa Cruz, CA, United States). After introducing the primer sequences in the application *in silico* PCR (www.genome.ucsc.edu, University of California, Santa Cruz, CA, United States), they showed only one product for each primer pair, confirming that they were specific and that all observed genes should correspond to the one sought.

Then, the position of the primers was searched in the mRNA. The forward and the reverse primers were located in different exons, ensuring that the amplified product was the mature transcript, after the alternative splicing. This requirement was studied using the application “BLAST/BLAT search” at www.ensembl.org (EMBL-EBI, Cambridge, United Kingdom).

The following forward and reverse primer sequences were used in RTQ-PCR assays to reverse-amplify the mRNA sequences of the indicated transcription factors, pro-inflammatory cytokines, and mediators and M2 signature markers. 18S rRNA was amplified as a house-keeping gene for normalization purposes:

RORγt Fw: 5′ GCA AGT CCT TCC GAG AGA 3′
Rev: 5′GTG TGG TTG TTG GCA TTG TAG 3′
FOXP3 Fw: 5′TGT TCG CCT ACT TCA GAA ACC AC 3′
Rev: 5′CTC CCT TCT CGC TCT CCA CTC 3′
TNF-α Fw: 5′ CGG GCA GGT CTA CTT TGG AG 3′
Rev: 5′ ACC CTG AGC CAT AAT CCC CT 3′
IL-6 Fw: 5′ TGA TGC ACT TGC AGA AAA CAA 3′
Rev: 5′ GGT CTT GGT CCT TAG CCA CTC 3′
iNOS Fw: 5′ CAC AGC AAT ATA GGA TCA TCC A 3′
Rev: 5′ GGA TTT CAG CCT CAT GGT AAA C 3′
TGF-β Fw: 5′CAC CGG AGA GCC CTG CAT A 3′
Rev: 5′ TGT ACA GCT GCC GCA CAC A 3′
YMI Fw: 5′GGA TGG CTA CAC TGG AGA AA 3′
Rev: 5′AGA AGG GTC ACT CAG GAT AA 3′
FIZZ Fw: 5′ CCC TTC TCA TCT GCA TCT C 3′
Rev: 5′ CAG TAG CAG TCA TCC CAG CA 3′
Mannose Receptor Fw: 5′CAA GGA AGG TTG GCA TTT GT
Rev: 5′ CCT TTC AGT CCT TTG CAA GC 3′
18S Fw: 5′AAC ACG GGA AAC CTC ACC C 3′
Rev: 5′ CCA CCA ACT AAG AAC GGC CA 3′

### Cytokine ELISA

IL-6, IL-17, and TNF-α serum concentrations were measured using ELISA Kits according to the manufacturer’s instructions (BD Biosciences OptEIA^TM^ and Biolegend). The reaction was detected by peroxidase-conjugated streptavidin, followed by incubation with hydrogen peroxide as a substrate and ABTS (Sigma Aldrich Co., St. Louis, United States) as a chromogen. Sample cytokine concentrations were interpolated from standard curves of recombinant IL-6, IL-17, and TNF-α. Absorbance readings were performed at 405 nm.

### Statistical Analysis

The Kaplan–Meyer test was used to analyze differences in mortality rates between groups. Differences between the infected, treated or untreated groups, for the several parameters were analyzed by one-way ANOVA. Comparison of every mean with every other mean was performed with the Tukey *post hoc* test. The Spearman rank-order correlation test was used to evaluate the correlation between IL-6 serum concentration or IL-6 mRNA expression and either EF or SF. Differences were considered statistically significant when *p* < 0.05. GraphPad Prism version 7 and the open source *R* Studio software was used for data analysis.

## Results

### Infection With K98 Induces Cardiac Dysfunction in IL-10 KO Mice

**FIGURE 1:**
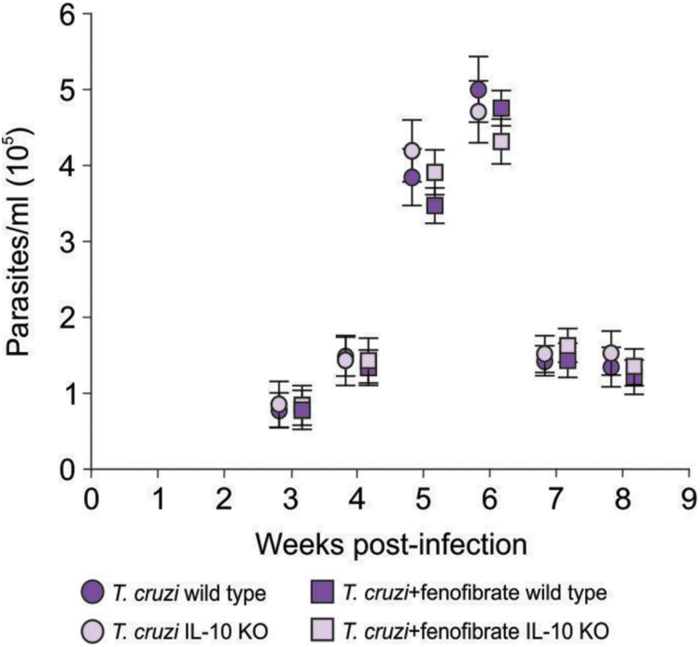
Parasitemia. Bloodstream parasites were quantified between 3- and 8-weeks pi, by the method of Pizzi modified by Brener, in infected and fenofibrate-treated IL-10 KO and WT. *N* = 7 mice per group. 3 replicate experiments. Point to point Student *t* test, mean ± SEM, *p* = NS.

**FIGURE 2:**
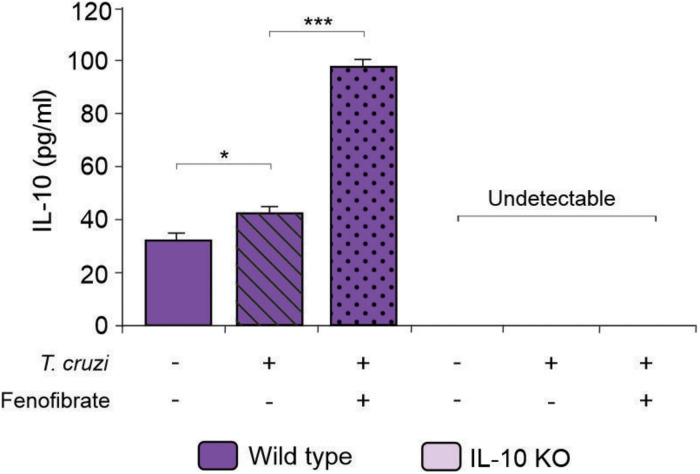
Determination of IL-10 concentration in sera. Concentration of IL-10 was measured by ELISA in sera from uninfected, *T. cruzi*-infected, and *T. cruzi*-infected fenofibrate-treated WT and IL-10 KO mice. Differences between groups were analyzed by ANOVA (mean ± SEM) followed by Tukey *post hoc* test. Differences were considered significant when *p* < 0.05. *N* = 7 mice per group, 3 replicate experiments. *p < 0.05 and ***p < 0.001.

IL-10 knockout and WT mice were infected with the K98 clone of *T. cruzi*, in order to evaluate the role IL-10 on the effects of fenofibrate treatment in an experimental model of Chagas disease. Parasitemia was analyzed during the course of infection in fenofibrate-treated and untreated mice. No differences were observed between groups. A peak was observed between weeks 5 and 6 pi, decreasing thereafter (Figure 1). Moreover, survival was unaffected in both mouse genotypes (data not shown). Thus, lack of IL-10 did not alter the susceptibility of mice to K98 infection. Serum samples were analyzed by ELISA to determine the levels of IL-10 in uninfected, *T. cruzi –*infected, and *T. cruzi* -infected and fenofibrate-treated WT mice. IL-10 KO mice were used as controls. As shown in Figure 2, IL-10 levels increased in the serum of WT mice upon infection with *T. cruzi* K98 clone. Moreover, fenofibrate treatment of infected WT mice further increased this cytokine. On the contrary, IL-10 was undetectable in sera of IL-10 KO mice.

**FIGURE 3:**
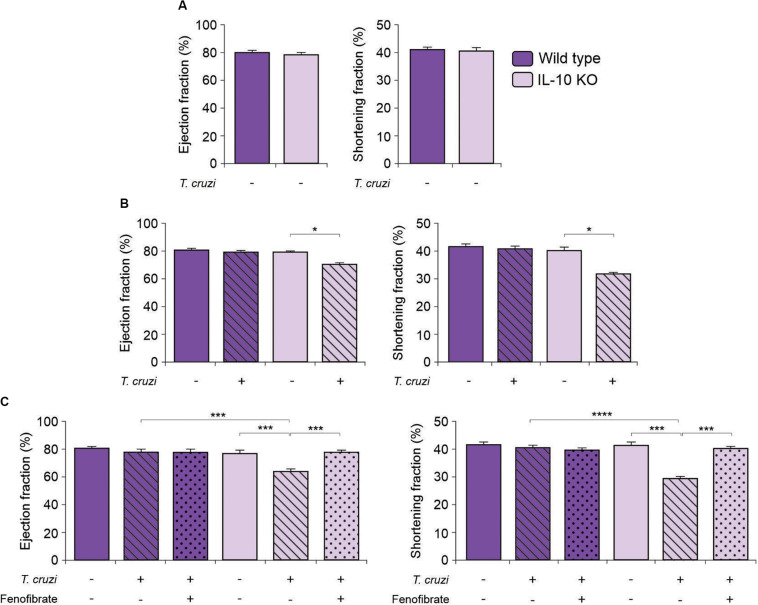
Echocardiographic studies in IL-10 KO and WT mice. The Ejection fraction and the Shortening fraction were measured by echocardiography in uninfected, *T. cruzi-*infected, and *T. cruzi-*infected fenofibrate-treated IL-10 KO and WT mice. **(A)** Ejection fraction and Shortening fraction measured before infection in IL-10 KO and WT mice. **(B)** Ejection fraction and Shortening fraction measured, at week 5, in uninfected and *T. cruzi-*infected IL-10 KO and WT mice. **(C)** Ejection fraction and Shortening fraction measured, at week 9 in uninfected, *T. cruzi-*infected and *T. cruzi-*infected and fenofibrate-treated (treatment finished) IL-10 KO and WT mice. Differences between groups were analyzed by Student *t* test (mean ± SEM) and ANOVA (mean ± SEM) followed by Tukey *post hoc* test. 7 mice *per* group, 3 replicate experiments. *p < 0.05; ***p < 0.001; and ****p < 0.0001.

In order to deepen into the role of IL-10 in the modulatory effects of fenofibrate, we tested whether there were differences in the echocardiographic parameters between IL-10 KO and WT mice. The EF and SF of uninfected IL-10 KO and WT mice did not show differences (Figure 3A). Then, IL-10 KO and WT mice were infected with the K98 myotropic clone of the CA-I strain. The IL-10 KO mice displayed echocardiographic alterations at week 5 pi (Figure 3B). On the other hand, echocardiographic parameters of *T. cruzi-*infected WT mice revealed no significant differences compared to controls (Figure 3B). After four weeks of treatment with fenofibrate, infected mice were analyzed by echocardiography (9 weeks post-infection). Restoration of EF and SF values to normal were observed in IL-10 KO mice (Figure 3C). As expected, no changes in the echocardiographic parameters were observed in WT mice (Figure 3C).

### Fenofibrate Has no Effects on Heart Inflammatory Infiltrates in IL-10 KO Mice

**FIGURE 4:**
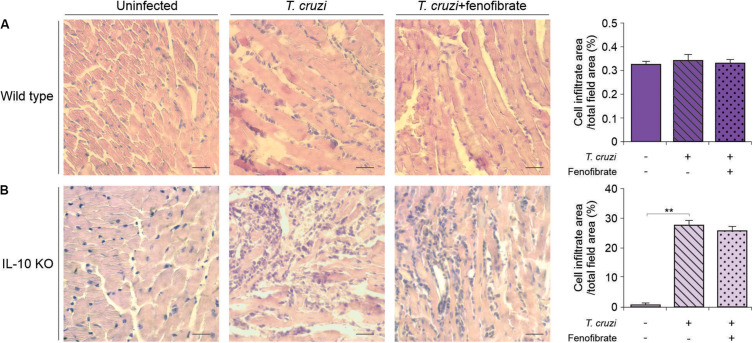
Inflammatory reaction in the heart. Histological sections of the heart of uninfected (left panel), *T. cruzi-*infected (center panel), and *T. cruzi-*infected fenofibrate-treated (right panel), WT **(A)** and IL-10 KO **(B)** mice. Bar graphs: infiltrate score quantification. Differences between groups were analyzed by ANOVA (mean ± SEM) followed by Tukey *post hoc* test. 7 mice *per* group, 3 replicate experiments. **p < 0.01.

**FIGURE 5:**
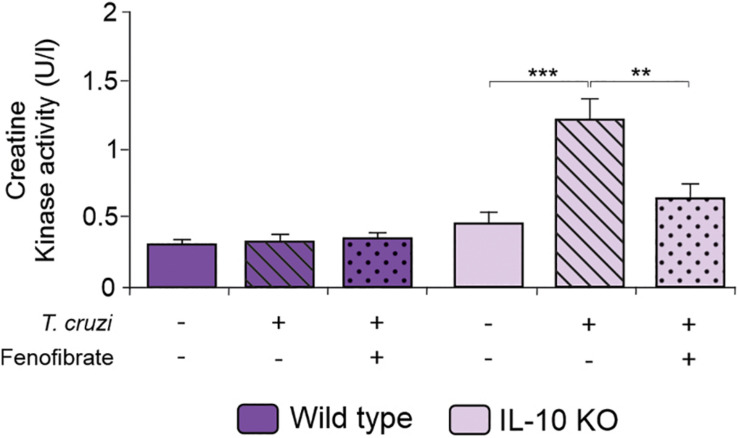
Serum Creatinine Kinase. CK activity was measured in the serum of uninfected, *T. cruzi-*infected, and *T. cruzi-*infected fenofibrate-treated WT and IL-10 KO mice using a colorimetric assay. Differences between groups were analyzed by Student *t* test and ANOVA followed by Tukey *post hoc* test. Differences between groups were analyzed ANOVA (mean ± SEM) followed by Tukey *post hoc* test. Differences were considered significant when *p* < 0.05. 7 mice *per* group, 3 replicate experiments. **p < 0.01 and ***p < 0.001.

We have previously reported that fenofibrate significantly reduces the extension of heart infiltrates in the mixed model of infection with bloodstream trypomastigotes of the K98 clone for 6 weeks, and re-infected with bloodstream trypomastigotes of the virulent RA strain (38). Then, we sought to determine whether, under IL-10 deficiency conditions, fenofibrate was able to promote a similar reduction using the single infection model, since IL-10 KO did not survive the co-infection infection (data no shown). As shown in Figure 4B the heart of infected IL-10 KO mice developed a significant interstitial inflammatory response, that fenofibrate was unable to control. On the other hand, WT mice developed mild heart infiltrates upon infection with the K98 clone, regardless of fenofibrate treatment (Figure 4A). Thus, these results lead to the conclusion that IL-10 is required to control the extent of the inflammatory infiltrate in the heart. CK levels were increased at week 9 pi in IL-10 KO mice compared to controls, and fenofibrate restored CK concentration to normal values. On the other hand, no changes were observed in WT mice (Figure 5).

### Fenofibrate Reduces Fibrosis in the Heart of IL-10 KO Mice Infected With *T. cruzi*

**FIGURE 6:**
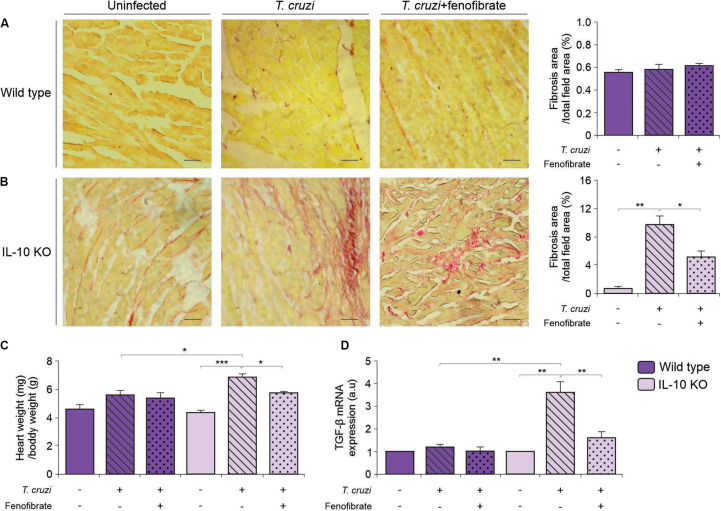
Heart remodeling and fibrosis. Whole hearts where perfused, excised, and weighted. Fibrosis was evaluated in heart sections of uninfected (left panels), *T. cruzi-*infected (center panels), and *T. cruzi-*infected fenofibrate treated WT **(A)** and IL-10 KO **(B)** mice were stained with picrosirius red. Bar graphs to the right: quantitative analysis of fibrosis. **(C)** Heart/body weight ratio was evaluated in uninfected, *T. cruzi-*infected, and *T. cruzi-*infected fenofibrate-treated IL-10 KO and WT mice. **(D)** The expression of TGF-β was determined by qRT-PCR in heart homogenates of uninfected, *T. cruzi-*infected and *T. cruzi-*infected fenofibrate-treated IL-10 KO and WT mice. Differences between groups were analyzed by ANOVA (mean ± SEM) followed by Tukey *post hoc* test. Differences were considered significant when *p* < 0.05. 7 mice *per* group, 3 replicate experiments. *p < 0.05; **p < 0.01; and ***p < 0.001.

Fibrosis is a hallmark of progressive heart remodeling during chronic *T. cruzi* infection. Since we found that fibrosis was limited upon treatment with fenofibrate in a previous study using a mixed *T. cruzi* infection model (38), heart sections were stained with picrosirius red to search for collagen deposits in the hearts of IL-10 KO mice infected with the K98 strain of *T. cruzi*. Interstitial fibrosis was observed in tissue sections of the myocardium. Remarkably, fenofibrate significantly reduced collagen deposits in infected IL-10 KO mice (Figure 6B). Interestingly, no fibrosis developed in K98-infected WT mice (Figure 6A). In agreement with these histopathological findings, the weight of *T. cruzi* infected hearts from IL-10 KO mice was significantly higher than that of WT mice. Moreover, while fenofibrate was able to reduce the heart weight of IL-10 KO significantly, no differences were found in WT mice. As a whole, weight reduction could be ascribed to the diminished fibrotic response upon fenofibrate treatment (Figure 6C).

We have previously shown that fenofibrate reduces heart fibrosis and that this phenomenon is accompanied by a decrease in the levels of the pro-fibrotic factors connective tissue growth factor (CTGF) and matrix metalloproteinase 9 (MMP 9) (38). Since TGF-b is a profibrotic factor upstream CTGF (45), we considered to study its expression at the mRNA level by qRT-PCR in this organ. TGF-β transcription increased upon infection of IL-10 KO mice, while no changes were observed in WT mice. The increased transcription was reduced as a result of fenofibrate treatment (Figure 6D).

### Fenofibrate Modulates Proinflammatory Mediators Independently of IL-10

**FIGURE 7:**
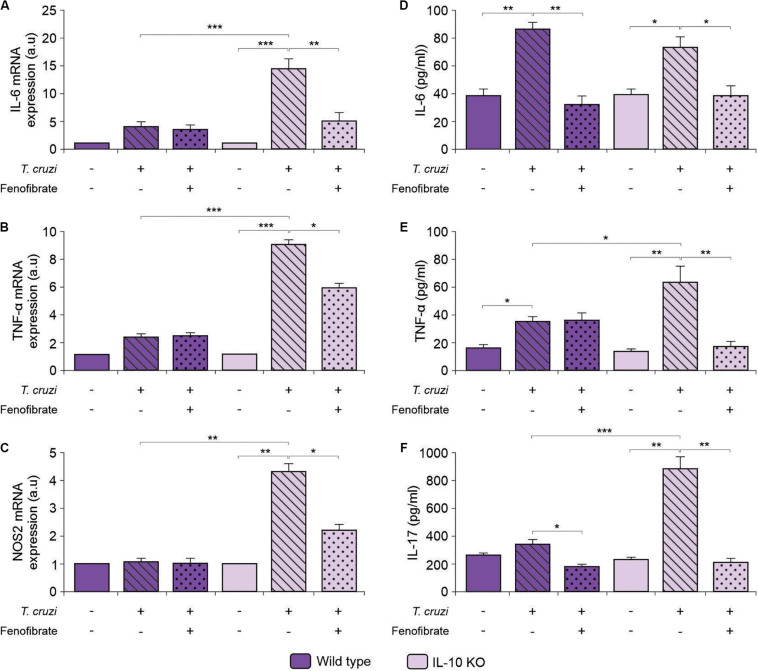
Assessment of the expression and concentration of proinflammatory mediators. Expression of IL-6 **(A)**, TNF-α **(B)**, and NOS2 **(C)** was determined by qRT-PCR in heart homogenates of uninfected, *T. cruzi-*infected, and *T. cruzi-*infected fenofibrate-treated WT and IL-10 KO mice. Concentration of IL-6 **(D)**, TNF-α **(E)**, and IL-17 **(F)** was measured in sera from uninfected, *T. cruzi-*infected, and *T. cruzi-*infected fenofibrate-treated WT and IL-10 KO mice. Differences between groups were analyzed by ANOVA (mean ± SEM) followed by Tukey *post hoc* test. Differences were considered significant when *p* < 0.05. 7 mice *per* group, 3 replicate experiments. *p < 0.05; **p < 0.01; and ***p < 0.001.

We have previously shown that proinflammatory mediators (NOS2, IL-6, and TNF-α) increase in the heart of mice after co-infection with *T. cruzi* strains, and that fenofibrate is able to modulate this response (38). To determine whether fenofibrate could exert the same effect in the absence of IL-10 in the single infection model, we assessed the levels of mRNA expression of the M1 markers IL-6, TNF-α and NOS2 in knock out mice. Infection increased their transcription, while treatment with fenofibrate reduced them significantly (Figures 7A–C, respectively). On the contrary, mRNA of these markers remained unchanged independently of the WT mice group (Figures 7A–C, respectively).

When the concentration of proinflammatory cytokines was assessed in sera of *T. cruzi*-infected IL-10 KO and WT mice, an increase in IL-6 (Figure 7D), TNF-α (Figure 7E), and IL-17 (Figure 7F) was observed in infected IL-10 KO mice, while treatment with fenofibrate reduced their concentration to control values.

**FIGURE 8:**
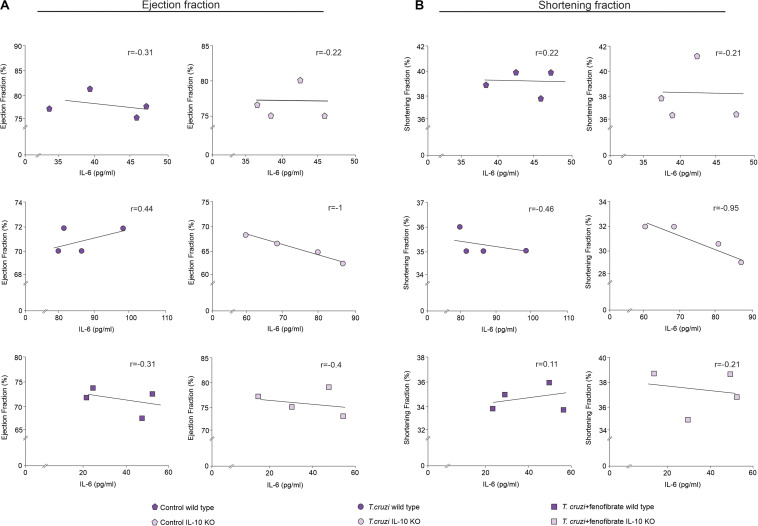
Relationship between cardiac disfunction and IL-6. Spearman rank-order correlation test between ejection fraction **(A)** or shortening fraction **(B)** and concentration of IL-6 in sera from uninfected, *T. cruzi*-infected, and *T. cruzi*-infected and fenofibrate-treated WT and IL-10 KO mice were made. Spearman correlation coefficient is reported for each of the correlations.

The correlation between IL-6 and cardiac dysfunction was assessed. As shown in Figure 8A, the increase of IL-6 in the sera of *T. cruzi*-infected IL-10 KO mice is strongly correlated with a decrease of either EF or SF. Moreover, the correlations approached zero upon treatment with fenofibrate. This was associated with the fact that this drug reduced the levels of IL-6 and reversed cardiac dysfunction. Besides, there is no correlation between these parameters in WT mice. The same results were observed when the correlation between IL-6 mRNA expression and the functional parameters was studied (Supplementary Figure 1).

### IL-10 Is Required by Fenofibrate to Promote an M1-to-M2 Bias in Heart Tissue

**FIGURE 9:**
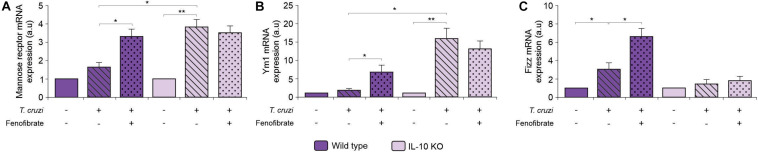
Effect of *T. cruzi* infection and fenofibrate treatment on the expression of M2 markers. The level of transcription of Mannose Receptor **(A)**, Ym1 **(B)**, and Fizz **(C)** was determined by qRT-PCR in heart homogenates from uninfected, *T. cruzi-*infected, and *T. cruzi-*infected fenofibrate-treated WT and IL-10 KO mice. Differences between groups were analyzed by ANOVA (mean ± SEM) followed by Tukey *post hoc* test. Differences were considered significant when *p* < 0.05. 7 mice *per* group, 3 replicate experiments. *p < 0.05 and **p < 0.01.

Wild-type and IL-10 KO mice were infected with the K98 clone of *T. cruzi* and the expression of mRNA of three M2 markers was analyzed at the end of fenofibrate treatment (9 weeks pi). As shown in Figure 9, fenofibrate promoted an increase in the amount of mannose receptor, YM1, and FIZZ, and mRNAs in the hearts of WT mice. On the other hand, infection with *T. cruzi* in the absence of IL-10 promotes the upregulation of these M2 markers that could not be further increased by fenofibrate. This suggests that IL-10 deficiency is circumvented by compensatory mechanisms to drive M1-to-M2 bias in *T. cruzi-*infected mice.

### Fenofibrate Modulates RORγt Expression Independently of IL-10

**FIGURE 10:**
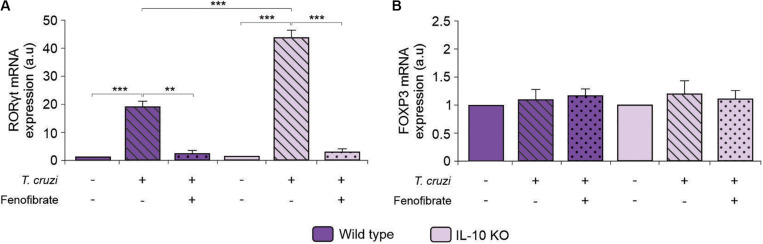
Expression of RORγt and FOXP3 in IL-10KO and WT mice. The level of transcription of RORγt **(A)** and FOXP3 **(B)** mRNA was determined by qRT-PCR in heart homogenates from uninfected, *T. cruzi-*infected, and *T. cruzi-*infected fenofibrate-treated WT and IL-10 KO mice. Differences between groups were analyzed by ANOVA (mean ± SEM) followed by Tukey *post hoc* test. Differences were considered significant when *p* < 0.05. 7 mice *per* group, 3 replicate experiments. **p < 0.01 and ***p < 0.001.

To deepen into the anti-inflammatory mechanism of fenofibrate in the model of CHD, we analyzed the capability of the PPARα ligand both to modify the expression of RORγt and FOXP3, since cells expressing these transcription factors are primarily committed to differentiate into Th17 and Treg cells, respectively. Consistent with the increase in serum IL-17 (Figure 7F), augmented expression of RORγt was observed in the heart of *T. cruzi*-infected mice, irrespectively of the expression of IL-10, while fenofibrate treatment could modulate RORγt expression (Figure 10A). Hence, these results would demonstrate that fenofibrate is able to regulate the differentiation of Th17 cells by precluding the expression of its master transcription factor. Moreover, no changes in the expression of FOXP3 were observed in IL-10 KO or in WT mice (Figure 10B).

## Discussion

Although the mechanisms that lead to the development of CCC are still intriguing due to the fact that not all infected patients develop heart anomalies, the relevance of parasite persistence as a trigger is presently acknowledged (8–10, 46–48). However, the understanding of the involvement of the different components of the inflammatory response and CCC is still unclear. This has led to attempts to treat the chronic stage of the disease solely based on the use of anti-parasitic drugs (49–51). Current evidence from experimental and human cases, however, shows almost invariably that the unrestrained inflammatory response is, at least in part, associated with the outcome of the disease. In this regard, several researchers have shown increased expression of pro-inflammatory cytokines in serum as well as in tissue in different experimental models of Chagas disease (52, 53), as well as associations between the increase in their concentration during the acute stage of the disease and the loss of contractile capability of myofibers during the chronic phase (54). Others have shown elevated pro-inflammatory cytokines in serum and heart sections of patients with CCC in comparison with patients with chronic asymptomatic infection (55–58). Interestingly, SNP polymorphisms in the genes encoding for pro-inflammatory cytokines, are involved in variations in serum concentration of the coded cytokines and might be related to the progression of CCC (59). These findings pose the question as to whether treatments based on the combination of drugs that modulate the inflammatory response in conjunction with the presently available anti-parasitic treatment, would be a better approach for the treatment of CCC and other chronic parasitic diseases with a significant inflammatory component. Taking all this into account, our group has led research on the effect of different PPAR agonists in the context of Chagas disease (38, 60–63), that bear in common anti-inflammatory properties besides their effect on carbohydrate (PPARγ) or lipid (PPARα) metabolism. We have recently shown that BALB/c mice co-infected with two *T. cruzi* strains and treated benznidazole, in combination with fenofibrate, leads to better heart function and reduction of pro-inflammatory cytokines, infiltrated heart cells, and fibrosis than mice untreated with the fibrate (38). While the exact mechanisms involving such improvements were not fully understood, some clues were derived from studies conducted in human beings. It has been reported that patients with asymptomatic Chagas disease express higher levels of IL-10 than CCC patients (59, 64, 65). Therefore, we sought to determine the role of IL-10 in the anti-inflammatory effects of fenofibrate. For this purpose, we took advantage of IL-10 KO mice. Treatment of these mice with fenofibrate alone ameliorated the cardiac function. This strongly suggests that the anti-inflammatory effects of this drug are, at least in part, independent of IL-10 and, secondly, unrelated to the parasite load. In fact, we have previously shown that fenofibrate does not alter parasitemia or tissue parasitism in a mixed infection murine model of chronic CHD (38).

Given the fact that fenofibrate might play different roles that would or would not depend on IL-10 expression, we assessed the levels of IL-10 in uninfected, *T. cruzi-*infected, and *T. cruzi -*infected and fenofibrate-treated WT and IL-10 KO mice. IL-10 increased upon *T. cruzi* infection in WT mice. Moreover, treatment of WT mice with fenofibrate increased IL-10 levels further. Notably, the absence of this cytokine in IL-10 KO mice, correlated with increased infiltrate extension in the heart.

These findings support the fact that IL-10 is required to modulate heart tissue infiltration and that, contrary to what happens in the mixed infection model of WT mice (38), fenofibrate is unable to exert this role in IL-10 KO mice. However, this effect may be probably hindered by the fact that heart infiltration is scarce due to the low virulence of the *T. cruzi* K98 clone, in the single strain infection model of WT mice.

The ongoing inflammatory response together with the local expression of pro-inflammatory mediators like IL-6, IL-17, TNF-α, and NOS2 contributes to tissue damage. Associated with this, we observed that, lack of IL-10 promotes severe cardiac dysfunction that correlates with increased IL-6 production. Moreover, fenofibrate is able to mitigate this effect (Figure 8). The inflammatory response subsequently drives tissue remodeling involving fibroblast proliferation and collagen deposition. In the experimental model analyzed in this work, fenofibrate was able to reduce the expression of pro-inflammatory mediators and fibrosis regardless of IL-10 expression. This phenomenon was associated with the capability of fenofibrate to reduce the transcription of TGF-β, which is the main regulator of fibrosis (66). Besides, it has been documented that this cytokine is involved in heart remodeling by altering the pattern of secretion of MMP, collagen, fibronectin, and other contributing fibrogenic mediators (67). Interestingly, heart remodeling involving fibrosis is associated with high TNF-α and TGF-β expression (68). Thus, fenofibrate would act in two arenas to preclude its pathological consequences, simultaneously reducing TGF-β and TNF-α and, as we show in this manuscript, these effects are independent of IL-10 production. Despite the fact that TGF-β transcription remains unaffected in WT, IL-10 might play a role favoring non-pathogenic heart remodeling, since it has been shown to promote M1-to-M2 macrophage polarization and hyaluronan degradation in a mouse model of myocardial infarction (34). Interestingly, while infected WT mice display a clear M1-to-M2 polarization that is further increased by fenofibrate, their IL-10 KO counterparts do not. Significantly, WT polarization was associated with the increase of mannose receptor and Ym1 expression, while FIZZ expression was unaffected. In this regard, Atochina et al. (69), reported that the glycan LNFPIII rapidly upregulates Arg1 and Ym1 on macrophages. However, LNFPIII-activated macrophages did not show upregulation of other alternatively activated macrophage markers, like FIZZ-1, MGL-1, and MMR *in vivo*. Similar results were shown by Osada et al. (70) upon infection of streptozotocyn-induced diabetic mice with *Schistosoma mansoni*. Since FIZZ-1 increases in WT mice in our model, we can hypothesize that IL-10 is required for FIZZ-1 upregulation. In our CHD model, IL-10 KO mice treated with fenofibrate do not display any M1-to-M2 bias, which is crucial to control the process of heart remodeling. However, under IL-10 deficiency conditions, fenofibrate is still able to reduce TGF-β transcription, thereby limiting collagen accumulation in heart tissue, as observed in the histological sections. Moreover, we found that the reduced expression of pro-inflammatory cytokines and TGF-β was associated with heart function amelioration. Similarly, a reduction in serum TGF-β concentration through pharmacological treatment associated with heart function improvement has been observed by others (71).

A recent work suggests that a worse clinical progression of CCC is associated with increased serum IL-17 and of alternative Th17 cell frequency (72). However, other authors have observed positive correlation between the levels of IL-17A and LV ejection fraction, suggesting IL-17A elevation is a good prognostic marker for CCC (73). In spite of this, treatment of C57Bl/6 mice with an anti-TNF-α monoclonal antibody (Infliximab^TM^) reduced the frequency of IL-17A^+^ and increased the frequencies of IL-10^+^ CD4 T cells, and also augmented IL-10^+^Ly6c^+^ and F4/80^+^ cells. This treatment ameliorated cardiac function and remodeling (53), thus suggesting a deleterious role of IL-17 in the mouse model.

Since we found that improvement of heart dysfunction and inflammatory response is associated with the inhibition of IL-17 and RORγt expression, it may be considered that fenofibrate treatment results in a better outcome through inhibition of Th17 cells. Besides IL-17, the expression of other pro-inflammatory cytokines, like IL-6, and TNF-α, decreases upon treatment with fenofibrate. In this regard, it has been reported that fenofibrate reduces colitis induced by dextran in IL-10KO C3H/HeJB mice, and this effect is due to the inhibition of IL-17 expression (74). Since both IL-6 and TGF-β are required for Th17 cell differentiation, it would be possible that fenofibrate precludes Th17 differentiation by limiting their concentration. In fact, the reduced concentration of IL-17 observed in serum of mice after fenofibrate treatment was associated with a significant reduction in the transcription of RORγt, the master regulator for Th17 cell differentiation. In this regard, the inhibition of *in vitro* differentiation of Th17 by fenofibrate treatment has been reported previously (75). Moreover, this inhibition has been shown to play a beneficial role in an experimental model of dextran-induced colitis (74) and in an experimental model of autoimmune myocarditis in mice and rats (76). Interestingly, FOXP3 expression remained unchanged after treatment with fenofibrate, suggesting that this drug mainly exerts its anti-inflammatory effects through the modulation of the pro-inflammatory branch. However, our results differ from those observed by Zhou et al. (77), showing the ability of fenofibrate to promote *in vitro* differentiation of Treg cells and those by Chang et al. (76), who show increased expression of FOXP3 in the hearts of rats with experimental autoimmune myocarditis, after fenofibrate treatment. This disagreement could be attributed to several factors, including the genetic background of mice used for these studies and the etiology of the pathological condition (autoimmune *vs.* infectious) analyzed.

**FIGURE 11:**
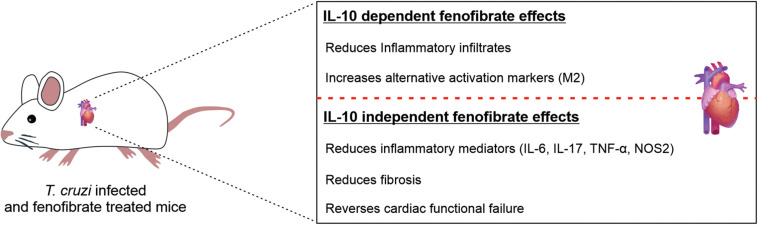
Fenofibrate exerts IL-10 dependent and IL-10 independent effects. The reduction of the inflammatory infiltrate extension and the increase in the expression of M2 markers are IL-10-dependent effects of fenofibrate, while the reduction of proinflammatory mediators expression, cardiac fibrosis and the restoration of cardiac function are IL-10 independent effects.

In conclusion, our results show that in the absence of IL-10, fenofibrate is able to ameliorate the heart function of *T. cruzi-*infected mice inducing the modulation of IL-17, among other cytokines, and preventing the development of fibrosis. Furthermore, our results emphasize that IL-10 is required to promote a healing profile in the heart, characterized by the expression of M2 markers (Figure 11). These findings may prove useful to design combined therapies that not only reduce the parasite burden but simultaneously lessen the noxious effects of the persistent inflammatory response.

## Data Availability Statement

The original contributions presented in the study are included in the article/Supplementary Material, further inquiries can be directed to the corresponding author/s.

## Ethics Statement

The animal study was reviewed and approved by Institutional Committee for the Care and Use of Laboratory Animals (CICUAL, Facultad de Medicina de la Universidad de Buenos Aires) in line with guidelines of the Argentinean National Administration of Medicines, Food and Medical Technology (ANMAT), Argentinean National Service of Sanity and Agrifoods Quality (SENASA), and also based on the US NIH Guide for the Care and Use of Laboratory Animals.

## Author Contributions

NG, GAM, and RG designed and supervised the experiments. CA provided the IL-10 knockout mice. JR, MD, FP, ÁC, and AP did the experiments. JR, MD, RG, NG, and GAM analyzed data. JR and FP contributed figures design. GAM and NG contributed to the writing of the manuscript. GAM and NG contributed to final approval of the version to be published. All authors contributed to the article and approved the submitted version.

## Conflict of Interest

The authors declare that the research was conducted in the absence of any commercial or financial relationships that could be construed as a potential conflict of interest.

## Supplementary Material

FIGURE S1 Relationship between cardiac disfunction and IL-6. Spearman rank-order correlation test between ejection fraction **(A)** or shortening fraction **(B)** and mRNA expression levels of IL-6 in sera from uninfected, *T. cruzi*-infected and *T. cruzi*-infected and fenofibrate-treated WT and IL-10 KO mice were made. Spearman correlation coefficient is reported for each of the correlations.
