# Supplementary figures and images for: IL-10-Dependent and -Independent Mechanisms Are Involved in the Cardiac Pathology Modulation Mediated by Fenofibrate in an Experimental Model of Chagas Heart Disease
Source: Front Immunol. 2020 Sep 24;11:572178. doi: 10.3389/fimmu.2020.572178 (PMC7541836; doi:10.3389/fimmu.2020.572178)

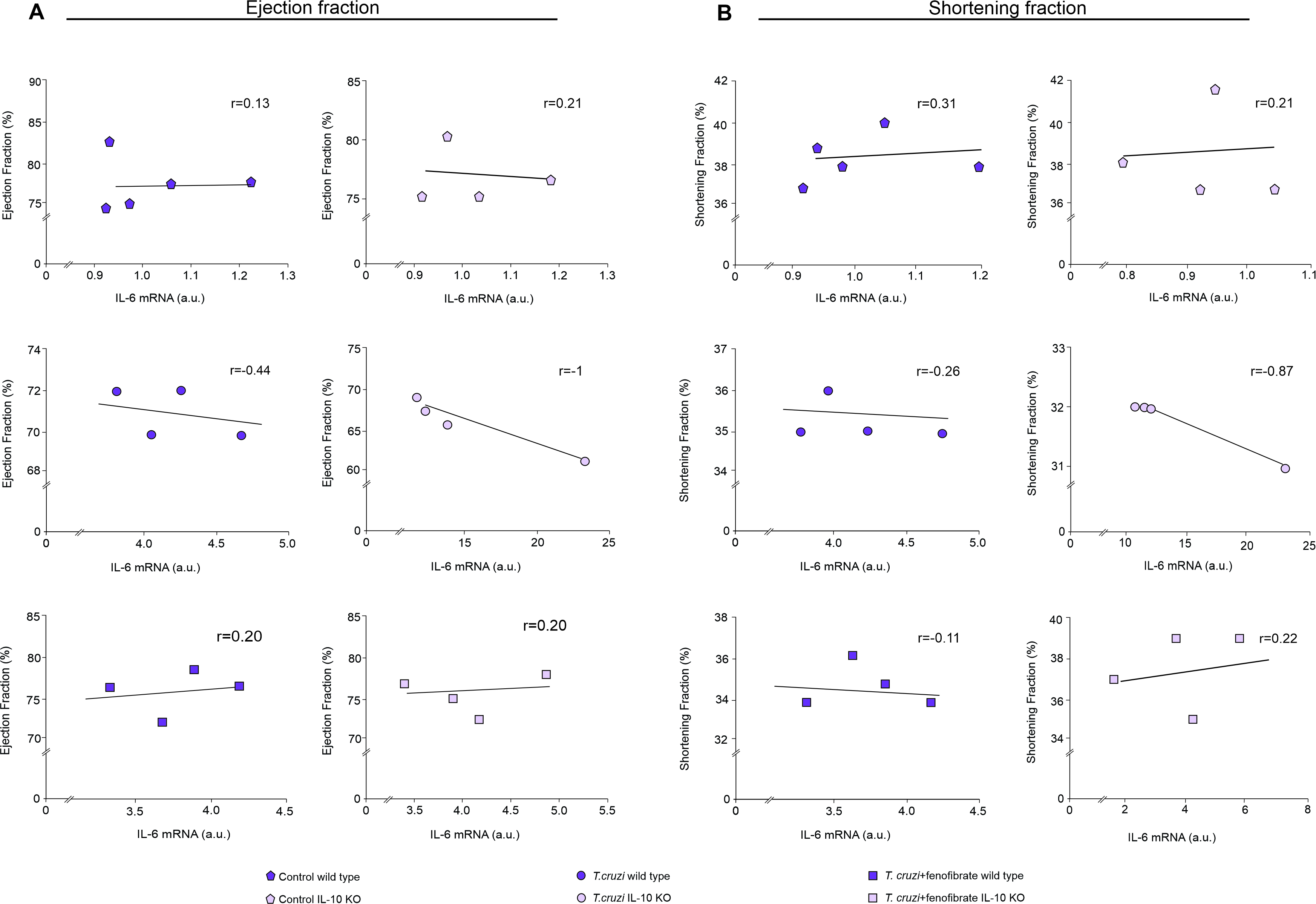

Supplement: FIGURE S1 — Relationship between cardiac disfunction and IL-6. Spearman rank-order correlation test between ejection fraction (A) or shortening fraction (B) and mRNA expression levels of IL-6 in sera from uninfected, T. cruzi-infected and T. cruzi-infected and fenofibrate-treated WT and IL-10 KO mice were made. Spearman correlation coefficient is reported for each of the correlations. [file Image_1.jpg]
